# Statistical Analysis of the Mechanical Behavior of High-Performance Polymers: Weibull’s or Gaussian Distributions?

**DOI:** 10.3390/polym14142841

**Published:** 2022-07-12

**Authors:** Yuri Boiko, Vyacheslav Marikhin, Lyubov’ Myasnikova

**Affiliations:** Laboratory of Physics of Strength, Ioffe Institute, 26 Politekhnicheskaya Str., St. Petersburg 194021, Russia; v.marikhin@mail.ioffe.ru (V.M.); liu2000@mail.ru (L.M.)

**Keywords:** high-performance polymers, mechanical properties, Weibull’s statistics, Gaussian statistics

## Abstract

This work addresses the following problem: which of the statistical approaches, Weibull’s or Gaussian, is more appropriate to correctly describe the statistical distributions of the mechanical properties of the high-performance polymer materials of different sample types (single or multifilament oriented fibers) and chain architectures (ultra-high-molecular-weight polyethylene, polyamide 6, or polypropylene)? Along with the routine mechanical properties such as strength, strain at break, and Young’s modulus, an apparent viscoelastic modulus and an apparent strain at break found when differentiating the stress–strain curves have been considered for the first time. For this purpose, a large sample number (50 in each series) has been tested. It has been shown that the values of the Weibull’s modulus (*m*) characterizing the data scatter were dependent both on the chain architecture and the sample type for the five elastic, viscoelastic and fracture characteristics analyzed. The Weibull’s model has been found to be more correct as compared to the Gaussian one. The different statistical approaches used for the analysis of the large arrays of the data are important for a better understanding of the deformation and fracture mechanisms of quasi-brittle and quasi-ductile high-performance polymer materials.

## 1. Introduction

High-performance oriented polymer materials are promising materials in various fields of application. One of the effective methods of their production is the drawing of isotropic low-strength polymers [1]. The initial lamellar crystallites in isotropic polymers are transformed in the course of orientation into rigid oriented fibrils due to the unfolding of the polymer chain folds in the initial lamellae. Such a discrete “structural re-arrangement” leads to a significant (by an order of magnitude or more) increase in the material important mechanical characteristics —Young’s (or elastic) modulus (*E*) up to 200 GPa and strength (*σ*) up to 6 GPa [1,2,3]. The advantage of such materials with respect to high-performance inorganic materials lies in their extremely high specific characteristics (related to the material density *ρ*). For instance, for a high-strength steel characterizing with very high values of *σ* = 1–2 GPa [4], the *σ*-to-*ρ* ratio, at a steel density of *ρ* ≈ 8 × 10^3^ kg/m^3^, is *σ*/*ρ* = 0.13–0.25 × 10^−3^ GPa·m^3^/kg while that for the ultra-oriented ultra-high-molecular-weight polyethylene (UHMWPE) gel fibers with *σ* = 6 GPa [2] and *ρ* ≈ 1 × 10^3^ kg/m^3^ attains *σ*/*ρ =* 6 × 10^−3^ GPa·m^3^/kg, i.e., it is 25–50 times greater than that for steel.

However, one of the drawbacks of the drawing process is the material embrittlement resulting in a sharp increase in a scatter of the measured mechanical properties. This behavior is due to the formation and development of surface microcracks at the final stages of orientational drawing, the so-called kink bands, which are initiators of the most intense crack growth at the deformation stages preceding the sample failure [1,2]. For this reason, in order to correctly estimate the average values of *σ*, *E*, and strain at break (*ε*_b_), *σ*_av_, *E*_av_, and *ε*_av_, which are important characteristics playing a key role in choosing the most appropriate fields for material applications, the number of identical samples to be tested should be increased markedly, from the generally accepted about five [5,6,7,8] to some dozens, or even hundreds [2,9,10,11,12,13,14,15,16,17,18,19,20,21,22]. In this case, the statistical distributions of mechanical properties can also be analyzed, and additional information obtained, for instance, on their conformity to a certain theoretical model (e.g., Gaussian [23], Weibull’s [9,10,11,12,13,14,15,16,17,18,19,20,21,22]). It can be helpful for a better understanding of the deformation and fracture mechanisms of materials and for making a more proper recommendation about their practical use. Actually, if the distribution of a measured dataset can be represented with a bell curve that is characteristic of the Gaussian (or normal) distribution, it implies that the data scatter is caused by the sum of many independent and equally weighted factors [23]. By contrast, if it obeys the Weibull’s statistics, it should have the shape of a linear plot in specific coordinates “lnln[1/(1 − *P*_j_)] − ln*σ*”, where *P*_j_ is the cumulative probability of failure, suggesting the dominant role of the surface cracks and their propagation across the sample cross-section in the sample fracture [2,3,4,5,6,7,8,9,10,11,12].

Earlier [2,3,15], it has been shown for high-performance polymer materials that the more correct type of the statistical distributions, Weibull’s or Gaussian, of *σ*, *E*, and *ε*_b_, and the values of the statistical Weibull’s modulus (*m*) characterizing the data scatter (an increase in *m* means a decrease in the data scatter) were dependent both on the chain chemical structure and the sample type (single or multifilament fibers). However, statistical characterization of other mechanical properties, besides *σ*, *E*, and *ε*_b_, has not been investigated yet, though it is helpful for a better understanding of the deformation and fracture mechanisms. In this respect, it is interesting to perform a detailed analysis of the stress–strain curves of such materials in order to estimate other important characteristics [24] in addition to those indicated above.

So, the goal of our study was to carry out a detailed analysis of the stress–strain behavior of a number of high-strength polymer materials in an attempt to estimate additional mechanical characteristics, besides widely used *σ*, *ε*_b_, and *E*, and to investigate the conformity of their statistical distributions to the Gaussian and Weibull’s models.

For this purpose, UHMWPE, polyamide 6 (PA 6), and polypropylene (PP) were chosen. The choice of these polymers was motivated by different chain architectures and conformations resulting in their different abilities to strengthen. Each of these polymers has been investigated by using two different oriented sample types: single or multifilament fibers. It was motivated by different statistical natures of these samples: “statistical origin” of a multifilament sample (including some hundreds of individual fibers) and “non-statistical origin” of a single fiber. The stress–strain behavior of these high-strength oriented polymer materials will be analyzed in more detail than usual (i.e., analysis of *σ*, *ε*_b_, and *E* only), and the statistical distributions of new mechanical characteristics estimated when analyzing the stress–strain curves will be compared with those of *σ*, *ε*_b_, and *E*.

It should also be noted that, along with drawing, some enhancement of the mechanical properties of polymer-based materials can be obtained by introducing the filler into a polymer matrix (see, e.g., [25,26,27]). However, despite the significant increase in the values of *σ* and *E* for such composite materials with respect to those of neat polymers, the former are still markedly lower (*σ* = 0.7 GPa, *E* = 17 GPa [25]) as compared to those of highly oriented polymers, in particular, of gel-cast UHMWPE fibers (*σ* = 6 GPa, *E* = 200 GPa [1,2,3]).

## 2. Weibull’s Statistics

Weibull’s statistics, proposed initially for the *σ* distribution analysis only, are based on the idea that the brittle sample fracture is controlled by the weakest (local) link [21] when the cumulative probability function *P*(*σ*) describing the probability of failure of identical samples at or below stress *σ* is given by:

*P*(*σ*) = 1 − exp [−(*σ*/*σ*_0_)*^m^*], (1)

where *m* is the so-called Weibull modulus (or shape parameter, a measure of data dispersion) and *σ*_0_ is the scale parameter (having the physical meaning of *σ*_av_). For performing the Weibull’s analysis, a set of test results should be converted into an experimental probability distribution by ordering them from the lowest strength to the highest ones. The *j-*th result in the set of *n* samples is assigned to a cumulative probability of failure (*P*_j_):

*P*_j_ = (*j* − 0.5)/*n*. (2)

By rearranging Equation (1), taking the logarithm of both sides twice and replacing *P*(*σ*) with *P*_j_, one obtains Equation (3):

lnln[1/(1 − *P*_j_)] = −*m*·ln*σ*_0_ + *m*·ln*σ*. (3)

Equation (3) represents a linear regression:

*y* = *a* + *bx*, (4)

where *y* = lnln[1/(1 − *P*_j_)], *b* is the *m*, *x* is the ln*σ*, and *a* = −*m*·ln*σ*_0_ is the curve intersection with the *y* axis. By estimating *m* as the tangent to the curve lnln[1/(1 − *P*_j_)] = f(ln*σ*) by using the standard procedure of the linear regression analysis, one can calculate *σ*_0_ by solving Equations (5) and (6):

ln*σ*_0_ = −*a*/*m*, (5)

*σ*_0_ = exp (−*a*/*m*). (6)

Earlier [15], it has been suggested to apply this approach not exclusively to *σ*, but to *ε*_b_ and *E* as well. So, by replacing *σ* and *σ*_0_ in Equations (1) and (3) by *ε*_b_ and *ε*_0_, *E* and *E*_0_, one obtains:

lnln[1/(1 − *P*_j_)] = *m*·ln*ε*_b_ − *m*·ln*ε*_0_, (7)

*ε*_0_ = exp (−*a*/*m*), (8)

lnln[1/(1 − *P*_j_)] = *m*·ln*E* − *m*·ln*E*_0_, (9)

*E*_0_ = exp (−*a*/*m*). (10)

Equations (7) and (9) can be reduced to Equation (4), by analogy with *σ*, where *x* = ln*ε*_b_ or ln*E*, and *a* = −*m*·ln*ε*_0_ or *a* = −*m*·ln*E*_0_.

In order to analyze the stress–strain behavior of high-strength oriented polymers in more detail than usual (analysis of *σ*, *ε*_b_, and *E*), the first derivative of the stress–strain curves should be calculated and specific points found (see [24]). In this work, the statistical analysis of other mechanical properties, besides *σ*, *ε*_b_, and *E*, found on the stress–strain curves will be performed for the high-strength single and multifilament fibers of PA 6 for the first time.

## 3. Materials and Methods

The following samples have been investigated: (i) lab-scale ultradrawn (a draw ratio of 120) single film threads of UHMWPE cast from decalin; (ii) commercial gel-spun multifilament fibers of UHMWPE (*Dyneema* SK60) consisting of some hundreds of single fibers kindly supplied by *DSM*, the Netherlands, and commercially available oriented single and multifilament fibers of (iii, iv) PA 6 and (v, vi) PP. In total, six sample series have been investigated. The lab-scale oriented single film threads were produced from an UHMWPE nascent powder with a viscosity-average molecular weight of 3 × 10^6^ g/mol synthesized at a Boreskov Institute of Catalysis *(*Novosibirsk, Russia). A 1.5-wt% UHMWPE solution in decalin was prepared at 160 °C under stabilization of 0.5 wt% of an anti-oxidant (di-*tert*-butyl-*p*-cresol). The UHMWPE gel films were produced by casting a polymer dilute solution into a Petri dish followed by quenching at ambient temperature. After drying, the gel was transformed into a xerogel in the form of films of a thickness of 100 μm. As-produced xerogel film was cut into 1-mm-wide strips which were subjected to a multi-stage hot-zone drawing on a home-made device equipped with a pin heater [1]. The commercial single and multifilament fibers of UHMWPE, PA 6 and PP were used as received.

The samples were subjected to tensile loading on an Instron tester at ambient temperature. Force–displacement curves were recorded at a cross-head speed of 10 mm/min and an initial distance between the tester clamps of 10 mm for the lab-scale UHMWPE single film threads (of a width of 0.4 mm and a thickness of ~2 μm), and at a strain rate of 200 mm/min and a distance between the tester clamps of 500 mm for all five other commercial samples: (i) multifilament fibers of UHMWPE (linear density *D* = 179 tex); (ii) single filament with a diameter *d* of 0.20 mm; (iii) multifilament fibers (*D* = 213 tex) of PA 6; (iv) single fiber (*d* = 0.17 mm); and (v) multifilament fibers of PP (*D* = 1087 tex). The measured stress–strain curves were analyzed and mechanical properties were estimated. For obtaining statistically proper results, 50 identical samples were tested for each of the six above-listed materials.

The statistical distributions of mechanical properties have been investigated by using the Gaussian [23] and Weibull’s models [21]. In the first case, the variation range of a property under analysis was divided into several intervals for each of which the number of measured values and their percentage in the total set of measurements were estimated. Thereafter, the histograms of the probability density function (PDF, the fraction of the values included in a given interval with respect to the total measured values) as a function of property value were constructed, and their conformity to the bell-shaped curve that is characteristic of the Gaussian distribution was investigated. In the second case, the Weibull’s plots lnln[1/(1 − *P*_j_)] = f [ln(*property*)] were constructed and the values of the scale parameter (e.g., *σ*_0_) and the shape parameter (or Weibull’s modulus) *m* were estimated.

## 4. Results

**Figure 1:**
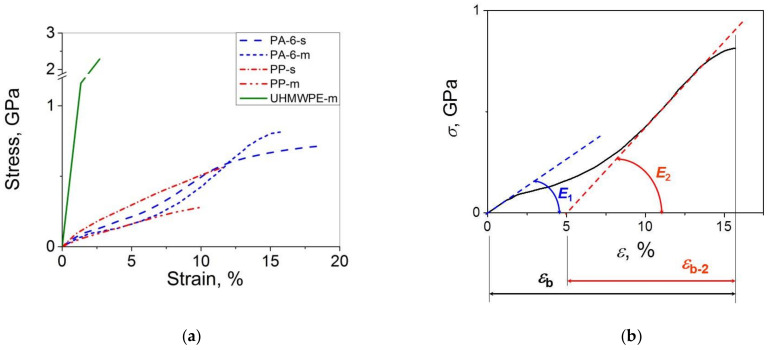
(**a**) Stress–strain curves for single (s) and multifilament (m) fibers of UHMWPE, PA 6 and PP recorded at a cross-head speed of 200 mm/min and an initial distance between the tester clamps of 500 mm; (**b**) Scheme of the stress–strain curve analysis for a PA 6 multifilament fiber.

Typical stress–strain curves for the high-performance single and multifilament fibers of PP, PA 6 and UHMWPE are shown in Figure 1. One can see that the curves for PP and UHMWPE demonstrate a monotonic increase in *σ* with *ε* until the sample fracture while those for PA 6 have a steeper curve portion at *ε* > 6% with respect to the initial curve portion at *ε* < 1%, indicating that additional strengthening takes place in the course of sample deformation. This behavior suggests that the strengthening potential has not been exhausted in the course of the drawing process, and the mechanical properties can be enhanced markedly even after an additional drawing of the samples by 10% only.

**Figure 2:**
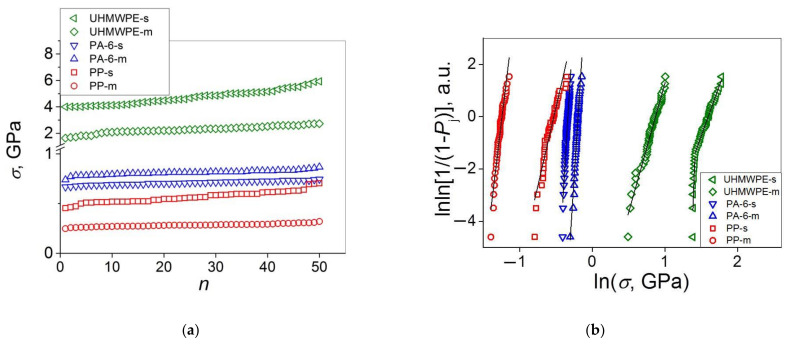

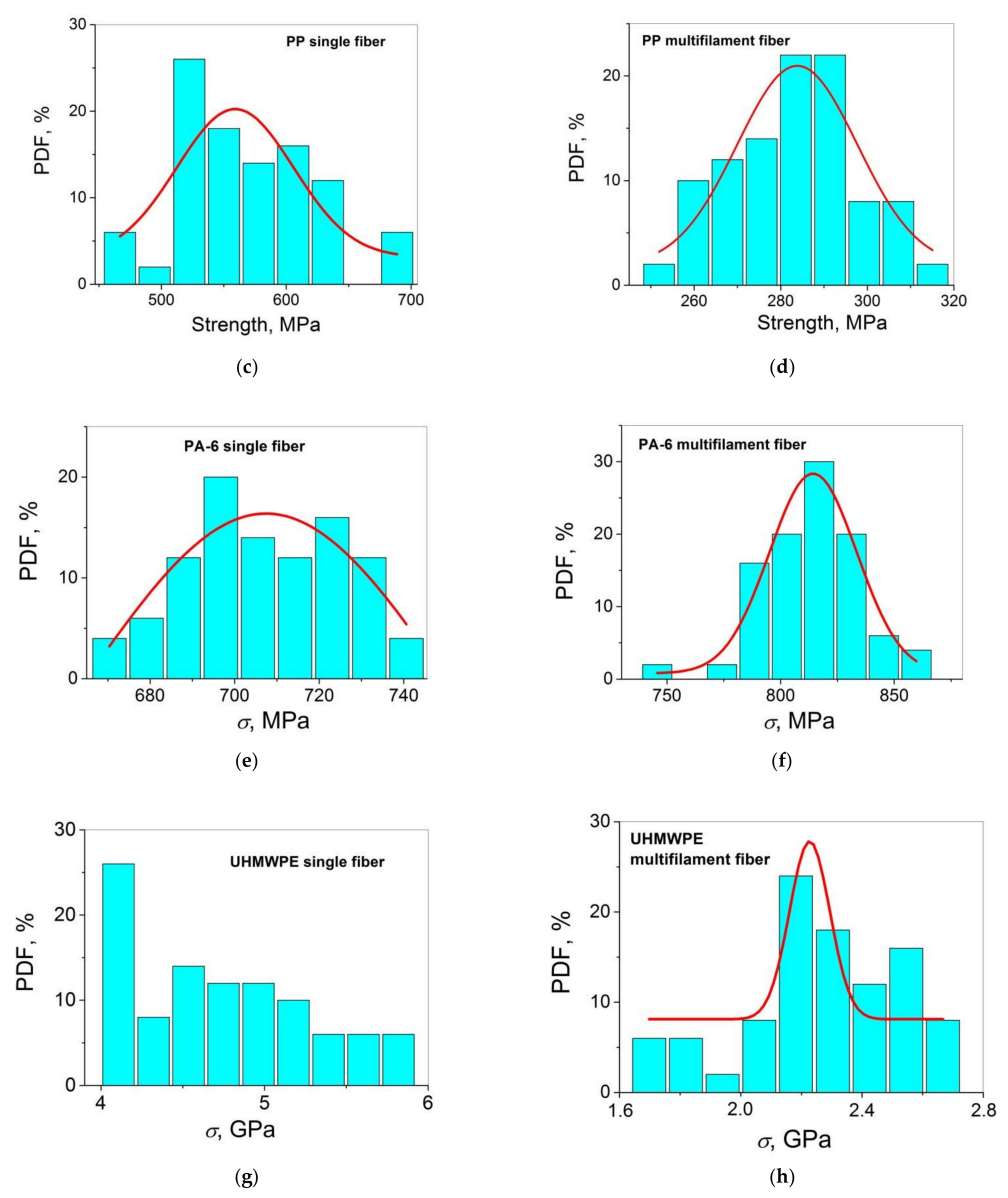
(**a**) Tensile strength *σ* as a function of sample number *n* in an ascending order for single and multifilament fibers of UHMWPE, PA 6 and PP; (**b**) Weibull’s plots for the data presented in (**a**), the solid lines are linear fits to the data sets; probability density function (PDF) vs. *σ* for: (**c**) UHMWPE single fibers, (**d**) UHMWPE multifilament fibers, (**e**) PA 6 single fibers, (**f**) PA 6 multifilament fibers, (**g**) PP single fibers, and (**h**) PP multifilament fibers; the bell curves PDF(*σ*) are the Gaussian fitting results.

Let us consider the stress–strain curve for the PA 6 multifilament for which this effect is more marked in more detail by taking the first derivative of the curve (see Figure 2). In this case, one observes two tangent lines corresponding to the Young’s modulus (*E*_1_) at small strains < 1% and an apparent viscoelastic modulus (*E*_2_) at large strains (*ε* > 10%). By extrapolating the second curve portion (*E*_2_) to *σ* = 0, the value of strain at break *ε*_b_ for this *ε* range (*ε*_b-2_) can be estimated. In this way, the second portion of the curve can be adjusted to a typical stress–strain behavior in the shape of a monotonic increase in *σ* with *ε* observed here for the PP and UHMWPE materials. By using this approach, one can also estimate, besides the ‘traditional’ mechanical properties such as *σ*, *ε*_b_ and *E*_1_, the values of *E*_2_ and *ε*_b-2_ and, thus, characterize the mechanical behavior of the PA 6 fibers more fully.

We first performed the statistical analysis of the characteristics which are relevant to all the six materials investigated, in particular, *σ*; and then *E*_2_ and *ε*_b-2_ found on the PA 6 stress–strain curves were considered. For this purpose, the *σ* values for each of the materials were arranged in an ascending order (see Figure 2a) and analyzed relative to their conformities to the Weibull’s and Gaussian statistical models. The results of this analysis are shown in Figure 2b, Figure 2c, Figure 2d, Figure 2e, Figure 2f, Figure 2g and Figure 2h, respectively. In addition, the Weibull’s analysis results are collected in Table 1. It is worth noting that the choice of the high-performance polymer materials under study allowed us to analyze their statistical mechanical behavior over a rather broad interval of strengths, from 0.2 to 6 GPa.

As follows from Figure 2b and Table 1, the constructed Weibull’s plots in the majority of cases (UHMWPE-m, PA 6-s, PA 6-m, PP-s, PP-m) represent single linear curves fitted with rather high reliability (root-mean-square deviation *R*^2^ > 0.95). For the UHMWPE single fibers, the data can be correctly fitted with two linear curves. However, 80% of the data pointing at higher strengths also demonstrate a high linear fitting reliability (*R*^2^ = 0.996). Besides, taking into consideration that the ratio of the parameter *σ*_0_ estimated from the Weibull’s plots (having the physical meaning of *σ*_av_) to *σ*_av_, *σ*_0_/*σ*_av_, is close to unity in all the cases considered, the results of the Weibull’s analysis performed seem to be correct.

As far as the Weibull’s modulus *m* is concerned, its value depends on: (i) the polymer’s chemical structure; (ii) the sample type; and (iii) the strength interval considered. In general, the *m* value for the multifilament is higher than the corresponding *m* value for the single fiber (carbon-chain UHMWPE and PP) or close to it (heterochain PA 6). For the ultra-high-strength UHMWPE single fibers, a markedly higher value of *m =* 74.0 was calculated for 20% of the samples with low strengths with respect to that of *m =* 7.7 calculated for 80% of the samples with high strengths. By contrast, a single linear curve with *m =* 9.9 is obtained when fitting the entire dataset of the UHMWPE multifilament. To put it differently, a higher *m* value for the UHMWPE multifilament (*m =* 9.9) as compared to that for the majority of the data for the UHMWPE single fiber (*m =* 7.7) indicates that the data scatter is narrower for the former. This observation suggests that the UHMWPE multifilament demonstrates a more reliable mechanical behavior as compared to that for the UHMWPE single fibers. Moreover, the observation of an excellent reproducibility (*m =* 74.0) of the low strength values (*σ* ≈ 4 GPa) for the latter ones indicates that the use of such materials is potentially more dangerous because there is an increased probability of fracture at *σ* < *σ*_av_ with respect to that for the UHMWPE multifilament. The fact that the *m* values for the UHMWPE and PP multifilament are higher with respect to the corresponding *m* values for the single fibers indicates that the role of critical cracks in the entire sample fracture is more important for the latter ones, which seems to be reasonable. Actually, the fracture of one filament should not necessarily result in the fracture of the entire multifilament sample including some tens or hundreds of single filaments.

The analysis of the strength data distributions on their conformity to the Gaussian model (see Figure 2c–h) has revealed that, for five of the six materials investigated, except the UHMWPE single fibers (see Figure 2g), the distribution histograms represent the bell-shaped curves suggesting that the measured data can be satisfactorily described in the framework of this model. It is also seen that, in general, (i) the fitting results are more correct for the three different multifilament samples than those for the corresponding single filaments, and (ii) for the materials with a lower strength.

When comparing the applicability of the Weibull’s and Gaussian models to the strength distributions of the high-performance polymer materials investigated, one can distinguish the statistical dualism of the strength distribution behavior for the materials with *σ* < 1 GPa. To put it differently, the four datasets for the PA 6 and PP materials investigated have been found to obey both the Weibull’s and Gaussian statistics. The conformity to the Gaussian statistics may be explained, on one hand, by their (though rather limited) plasticity (*ε*_b_ = 10–20%) preserving their brittle fracture. On the other hand, they do not undergo yielding (yield points are absent on the stress–strain curves—see Figure 1a). So, they can also be treated as quasi-brittle materials for which the Weibull’s statistics were proposed initially. The UHMWPE materials investigated characterized with smaller values of *ε*_b_ < 5% are referred to as quasi-brittle materials. So, it is not surprising that their strength distribution behaviors are in good accord with the Weibull’s model. The fact that the Gaussian model does not always work properly for these materials, in particular for the UHMWPE single fibers, indicates that the data *scatter* is not caused by the sum of many independent and equally weighted factors [23]. Rather, it is controlled by weakest links and a crucial role of surface cracks in the sample fracture, i.e., the key factors on which the Weibull’s statistics are based [2,21].

**Figure 3:**
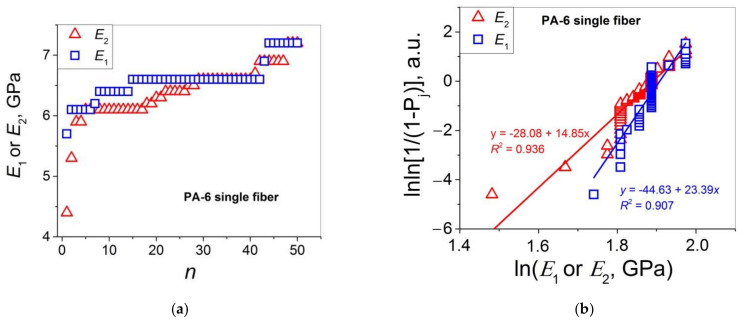

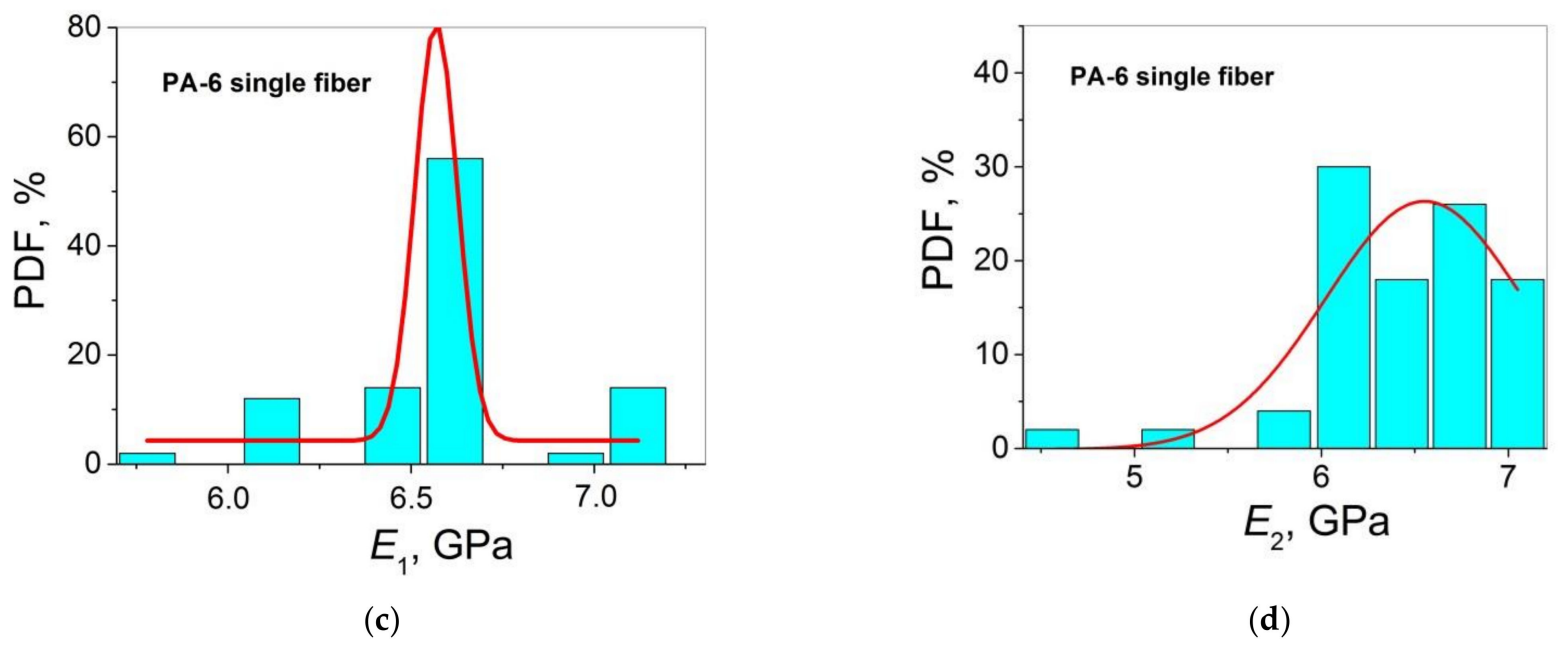
(**a**) Moduli *E*_1_ and *E*_2_ as functions of sample number *n* in an ascending order for PA 6 single fibers; (**b**) Weibull’s plots for the data presented in (**a**); PDF vs. *E*_1_ (**c**) and *E*_2_ (**d**) for the data presented in (**a**); the bell curves are the Gaussian fitting results.

**Figure 4:**
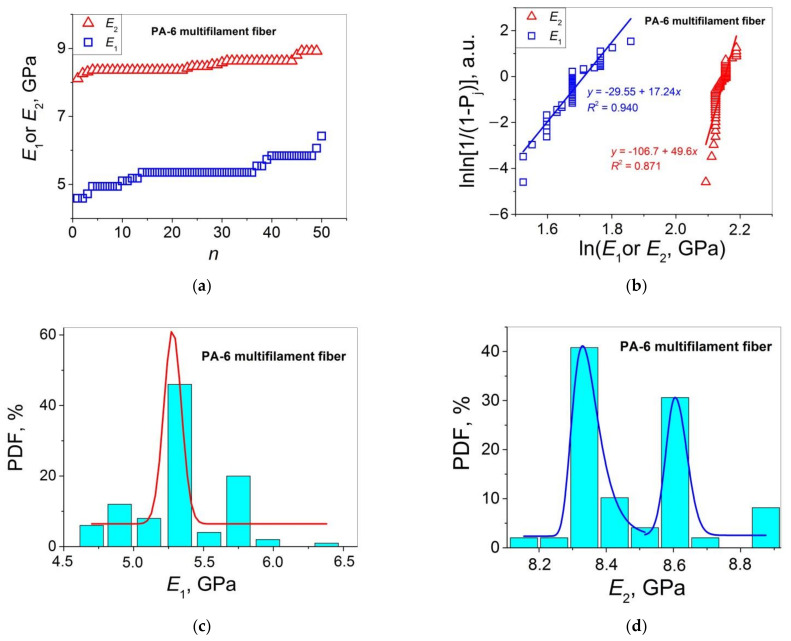
(**a**) Moduli *E*_1_ and *E*_2_ as functions of sample number *n* in an ascending order for PA 6 multifilament fibers; (**b**) Weibull’s plots for the data presented in (**a**); PDF vs. *E*_1_ (**c**) and *E*_2_ (**d**) for the data presented in (**a**); the bell curves are the Gaussian fitting results.

**Figure 5:**
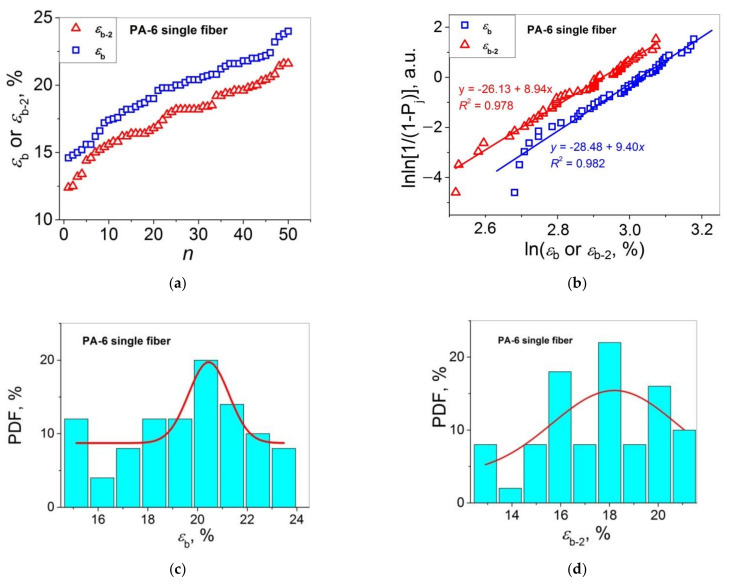
(**a**) Strains at break *ε*_b_ and *ε*_b-2_ as functions of sample number *n* in an ascending order for PA 6 single fibers; (**b**) Weibull’s plots for the data presented in (**a**); PDF vs. *ε*_b_ (**c**) and *ε*_b-2_ (**d**) for the data presented in (**a**); the bell curves are the Gaussian fitting results.

**Figure 6:**
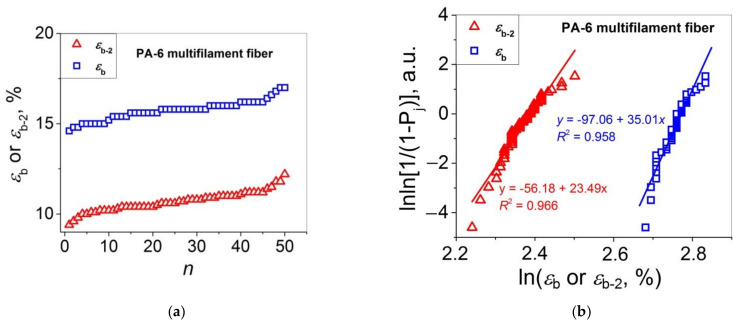

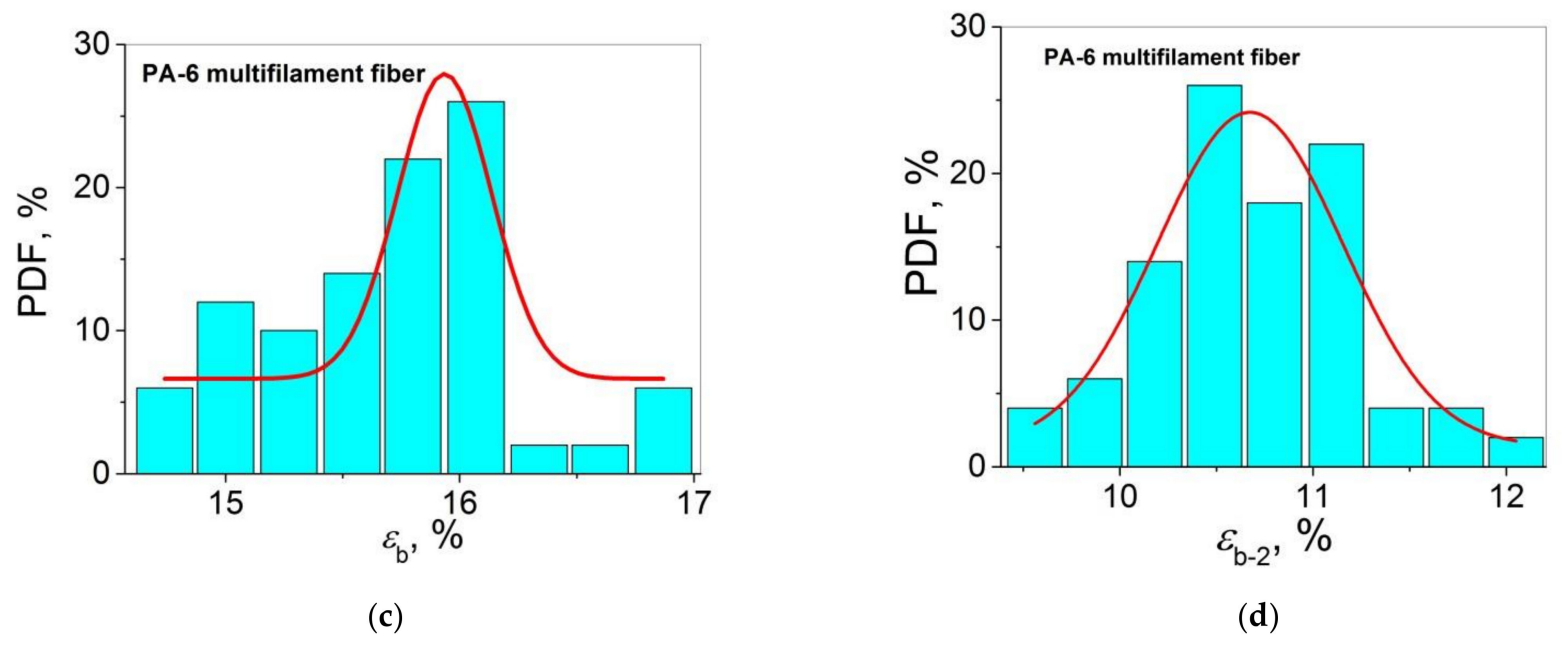
(**a**) Strains at break *ε*_b_ and *ε*_b-2_ as functions of sample number *n* in an ascending order for PA 6 multifilament fibers; (**b**) Weibull’s plots for the data presented in (**a**); PDF vs. *ε*_b_ (**c**) and *ε*_b-2_ (**d**) for the data presented in (**a**); the bell curves are the Gaussian fitting results.

Let us turn to the statistical analysis of the stress–strain curves of the two PA 6 materials under study by comparing the distributions of *E*_1_ (=*E*) and *E*_2_, and *ε*_b_ and *ε*_b-2_. The results of this analysis are presented in Figure 3 (*E*_1_ and *E*_2_ for single fibers), Figure 4 (*E*_1_ and *E*_2_ for multifilament fibers), Figure 5 (*ε*_b_ and *ε*_b-2_ for single fibers), and Figure 6 (*ε*_b_ and *ε*_b-2_ for multifilament fibers), and are collected in Table 2.

Let us apply the Weibull’s and Gaussian models to describe the statistical distributions of the values of *E*_2_ and *ε*_b-2_ presented in Figure 3a and Figure 4a, and Figure 5a and Figure 6a, respectively, in the same way as above for *E*_1_ and *ε*_b_. For this purpose, the values of *ε*_b_, *ε*_0_, *E*, and *E*_0_ in Equations (7)–(10) should be replaced by the corresponding values of *ε*_b-2_, *ε*_2-0_, *E*_2_, and *E*_2-0_. As a result, one obtains Equations (11)–(14) that will be used for the Weibull’s analysis of *E*_2_ and *ε*_b-2_ below.

lnln[1/(1 − *P*_j_)] = *m*·ln*ε*_b-2_ − *m*·ln*ε*_2-0_, (11)

*ε*_0_ = exp (−*a*/*m*), (12)

lnln[1/(1 − *P*_j_)] = m·ln*E*_2_ − m·ln*E*_2-0_, (13)

*E*_2-0_ = exp (−*a*/*m*). (14)

As follows from Figure 3a, the values of *E*_1_ and *E*_2_ for the PA 6 single fibers are close, indicating that the effect of the deformation strengthening is not characteristic of this material. These data are represented in Weibull’s coordinates “lnln[1/(1 − *P*_j_)] − ln(*property*)” in Figure 3b. The Weibull’s modulus values calculated from the tangents of these plots *m*(*E*_1_) = 23.4 and *m*(*E*_2_) = 14.9 indicate that that the scatter data for *E*_2_ are broader than that of *E*_1_. The data analysis in the framework of the Gaussian model shows that the bell-shaped curves can be received by computer fitting of the histograms for both *E*_1_ and *E*_2_ (see Figure 3c and Figure 3d, respectively). However, the curve obtained for *E*_1_ with a well-defined maximum is more symmetric while that for *E*_2_ demonstrates a rather diffuse maximum shifted to higher *E*_2_ values. Hence, the Gaussian model describes the distribution of *E*_1_ more correctly as compared to that of *E*_2_. By contrast, the Weibull’s model describes more correctly (*R*^2^ = 0.936; for the *E*_1_ distribution, *R*^2^ = 0.907) the *E*_2_ distribution. These two observations may be explained as follows. On one hand, the *E*_2_ value is calculated for the strains > 0.5*ε*_b_. In this strain range, the onset of the local fracture processes (chain scission, interfibrillar slipping) is expected. Hence, this mechanical property ‘approaches’ the fracture properties such as *σ* and *ε*_b_ for which the Weibull’s model works properly [2,3,4,5,6,7,8,9,10,11,12,13,14,15]. On the other hand, the *E*_1_ value characterizes the very beginning of the sample deformation. At this deformation stage, the contribution of the fracture processes such as covalent bond rupture and crack propagation is negligible. Therefore, the validity of the Weibull’s model in this case is less appropriate than the Gaussian one. Therefore, it may be concluded that the *E*_1_ data scatter is controlled by many independent and equally weighted factors, as expected in the framework of the Gaussian model.

Let us consider variations of the same mechanical properties for the PA 6 multifilament fibers. For this material, the *E*_2_ values are twice as high compared to the *E*_1_ values (see Figure 4a). This means that a significant improvement in the material rigidity takes place with an increase in *ε*. The Weibull’s modulus values calculated from the plots of Figure 4b are 17.2 and 49.6 for *E*_1_ and *E*_2_, respectively, i.e., the observed difference by a factor of three is rather large, indicating that the data scatter of *E*_2_ is significantly smaller as compared to that of *E*_1_. It may be indicative of the development of a more uniform structure in the course of deformation. As far as the Gaussian analysis is concerned, the histograms PDF(*E*_1_) and PDF(*E*_2_) presented in Figure 4c and Figure 4d, respectively, show that the *E*_1_ distribution can be fitted satisfactorily with a bell curve. By contrast, two maxima with rather high PDF values of 40 and 30% are observed on the *E*_2_ histograms, indicating that the *E*_2_ distribution in the framework of the Gaussian mode is bimodal and, hence, it has a rather complicated character.

Let us analyze the distributions of *ε*_b_ and *ε*_b-2_. The datasets *ε*_b_(*n*) and *ε*_b-2_(*n*) for the PA 6 single fibers presented in Figure 5a are plotted in the Weibull’s coordinates in Figure 5b. As one can see, the two linear fit curves are symbatic, and they are characterized with rather close *m* values for *ε*_b-2_ and *ε*_b_ (*m* = 8.94 and 9.40, respectively). The analysis of these data using the Gaussian model (see Figure 5c,d) shows that the results received are arbitrary. Therefore, the Weibull’s model seems to be more appropriate for describing correctly the statistical distributions of *ε*_b-2_ and *ε*_b_ for the PA 6 single fibers in comparison with the Gaussian model. Taking into consideration that the Weibull’s model has been proposed for a fracture property (*σ*), this behavior seems to be reasonable. For the PA 6 multifilament samples, one observes behavior similar to that for the PA 6 single fiber samples: symbatic curves *ε*_b_(*n*) and *ε*_b-2_(*n*) (see Figure 6a) and close tangents of the linear fit curves lnln[1/(1 − *P*_j_)] = f(ln*ε*_b_) and lnln[1/(1 − *P*_j_)] = f(ln*ε*_b-2_): *m* = 35.0 and 23.5, respectively.

It should be noted that the Weibull’s analysis carried out for the deformation characteristics considered is correct for the two sample types since it is characterized with *R*^2^ ≥ 0.98 and *R*^2^ ≥ 0.96 for the single and multifilament PA 6 fibers, respectively. However, in contrast to the Gaussian distributions of *ε*_b_ and *ε*_b-2_ for the PA 6 single fibers, the fitting results for the PA 6 multifilament, in particular, the curve for *ε*_b-2_, using the Gaussian model seem to be correct. The difference in the Gaussian distributions of *ε*_b_ and *ε*_b-2_ revealed for the two PA 6 sample types may be explained by the statistical nature of one multifilament sample (including about 200 individual fibers) in comparison with one fiber in the single fiber sample. To put it differently, the test results for the multifilament sample are substantially more verified statistically which is important for obtaining a proper Gaussian distribution.

From the data collected in Table 2, it follows that all the values of the ratio of the scale parameter to the average value of the corresponding mechanical property *p*_0_*/p*_av_ (*E*_2-0_*/E*_2-__av_, *ε*_2-0_*/ε*_2-__av_, *E*_1-0_*/E*_av_, *σ*/*σ*_av_, and *ε*_0_*/ε*_av_) for the two PA 6 sample types investigated are close to unity (*p*_0_*/p*_av_ ≈ 1), indicating that the Weibull’s analysis carried out is correct for all five mechanical properties investigated. In order to estimate the effect of the deformation strengthening at the second portion of the stress–strain curve, let us compare the statistically correct values of *E*_1_ and *E*_2_, i.e., the values of *E*_1-0_ and *E*_2-0_, respectively, calculated from the corresponding Weibull’s plots. Since the ratios *E*_2-0_/*E*_1-0_ for the single and multifilament PA 6 fibers are 0.98 and 1.55, respectively, this means that the slope of the second portion (at *ε* > 6%) of the stress–strain curve for the PA 6 multifilament is steeper than the initial portion (at *ε* < 1%) while those for the single fibers are almost equal. This suggests a marked effectiveness of strengthening of the PA 6 multifilament. It should also be noted that the applicability of the Weibull’s model is more correct for a fracture property (*ε*_b-2_, *R*^2^ > 0.96) with respect to a viscoelastic property (*E*_2_, *R*^2^ < 0.94). This behavior seems to be reasonable since this model has been proposed initially for a fracture property (*σ*).

Finally, let us compare the *m* values estimated for the two PA 6 sample types investigated (see Table 3) as the *m* ratios for the multifilament to those for the single fiber.

It is seen that these ratios are rather close for *σ* and, to a lesser extent, for *E*_1_ while they are markedly higher for the multifilament, by a factor of 3 to 4, for *E*_2_, *ε*_b_, and *ε*_b-2_. This means that, in general, the data scatter for the multifilament is substantially smaller than that for the single fiber. This behavior may be explained by an extremely large number of the fractured single fibers per one test for the multifilament sample including 200 single fibers with respect to the single filament sample (i.e., 200 against 1), making the results for the former more appropriate statistically which is reasonable.

## 5. Conclusions and Future Outlook

The analysis of the stress–strain behavior of the six high-performance polymer materials differing in the chain architecture (UHMWPE, PA 6 and PP) and the sample type (single and multifilament fibers) has revealed rather extended near-linear portions only on the curves of the two PA 6 samples investigated at *ε* > 5% characterized with a tangent equal to or larger than the tangent of the initial stress–strain portion characterizing the Young’s modulus *E*. By differentiating these curves, the two additional characteristics, the apparent modulus *E*_2_ and the apparent strain at break *ε*_b-2,_ have been estimated. A marked effect of strengthening of the PA 6 multifilament in the course of tensile testing (*E*_2_ > *E*_1_) has been found, indicating that the enhancement of the mechanical properties of this material proceeded more effectively than that of the PA 6 single fibers (*E*_2_ ≈ *E*_1_). It has been shown that the Weibull’s model is more correct for describing the statistical distributions of the mechanical properties of the high-strength PA 6 fibers than the Gaussian one. It has been shown that the data scatter for some of the mechanical properties (*E*_2_, *ε*_b_, and *ε*_b-2_), including those estimated, for the first time, by differentiating the stress–strain curves (*E*_2_ and *ε*_b-2_), is substantially smaller for the multifilament than that for the single fibers. This behavior is due to a substantially larger (by two orders of magnitude) number of the fractured single fibers per test in the multifilament samples as compared to the single fiber, making the results received for the former substantially more proper statistically.

Based on the results obtained in this work and earlier [2,3,15], we suppose that it is necessary to use both the Weibull’s and Gaussian models for the correct statistical characterization of the mechanical properties of the high-performance polymer materials of various chemical natures, fragilities and ductilities. The strength distribution behavior investigated over a wide range of strengths of 0.2 to 6 GPa has been shown to depend both on the chain architecture and the sample type. The notable deviations from the standard Weibull’s and Gaussian distributions have been observed for the ultra-strength UHMWPE single fiber. This suggests that the final drawing stages, up to the high draw ratio of 120, were accompanied by the significant structural damage.

Further in-depth analysis of the statistical distributions of the mechanical properties of high-performance polymer materials can be performed by involving the other statistical approaches such as the chi-square, Kolmogorov–Smirnov and D’Agostino–Pearson tests [28].

## Figures and Tables

**Table 1:** Results of Weibull’s analysis of the strength distribution of high-performance single (s) and multifilament (m) fibers of UHMWPE, PA 6 and PP.

Sample	*y* = *a* + *bx*	*R* ^2^	*m*	*σ*_0_, GPa	*σ*_av_, GPa	*σ*_0/_ *σ*_av_
UHMWPE-s	*y* = −106.12+ 73.98*x* ^1^ *y =* −12.28 + 7.69*x* ^1^	0.856 0.996	73.98 7.69	4.20 4.96	4.70 4.70	0.89 1.06
UHMWPE-m	*y* = −8.62 + 9.89*x*	0.987	9.89	2.39	2.23	1.07
PA 6-s	*y* = 15.14 + 45.32*x*	0.976	45.32	0.72	0.71	1.02
PA 6-m	*y* = 8.44 + 43.41*x*	0.982	43.41	0.82	0.81	1.02
PP-s	*y* = 6.34 + 12.02*x*	0.954	12.02	0.59	0.57	1.04
PP-m	*y* = 28.54 + 23.02*x*	0.973	23.02	0.29	0.28	1.04

^1^ Approximation with two linear portions.

**Table 2:** Results of Weibull’s distribution analysis of various mechanical properties (*p*) of single and multifilament fibers of PA 6.

Property, *p*	Fiber Type	*y* = *a* + *bx*	*R* ^2^	*m*	*p*_0_	*p*_av_	*p*_0_ */p*_av_
*E*_1_	single	*y* = −44.63 + 23.39*x*	0.907	23.39	6.75 GPa	6.60 GPa	1.02
*E*_1_	multifil.	*y* = −29.55 + 17.24*x*	0.940	17.24	5.56 GPa	5.38 GPa	1.03
*E*_2_	single	*y* = −28.08 + 14.85*x*	0.936	14.85	6.63 GPa	6.40 GPa	1.04
*E*_2_	multifil.	*y* = −106.68 + 49.55*x*	0.871	49.55	8.62 GPa	8.56 GPa	1.01
*σ*	single	*y* = 15.14 + 45.32*x*	0.936	45.32	0.72 GPa	0.71 GPa	1.02
*σ*	multifil.	*y* = 8.44 + 43.41*x*	0.982	43.41	0.83 GPa	0.81 GPa	1.02
*ε*_b_	single	*y* = −28.48 + 9.40*x*	0.982	9.40	20.7%	19.6%	1.06
*ε*_b_	multifil.	*y* = −97.06 + 35.01*x*	0.958	35.01	16.0%	16.1%	0.99
*ε*_b-2_	single	*y* = −26.13 + 8.94*x*	0.978	8.94	18.6%	17.6%	1.06
*ε*_b-2_	multifil.	*y* = −56.18 + 23.49*x*	0.966	23.49	10.9%	10.7%	1.02

**Table 3:** Comparison of the Weibull’s modulus *m* for single and multifilament PA 6 fibers for some mechanical properties.

Mechanical Property	*σ*	*E*_1_	*E*_2_	*ε*_b_	*ε*_b-2_
*m*(multifilament)/*m*(single)	0.96	0.74	3.34	3.72	2.63

## Data Availability

Not applicable.
